# Cross clamping of the supraceliac aorta is effective for bleeding control in ruptured giant splenic artery pseudoaneurysm when proximal and distal control of the splenic artery is not possible: a case report

**DOI:** 10.1590/1677-5449.202102102

**Published:** 2022-09-30

**Authors:** Venu Bhargava Mulpuri, Prashanth Gurijala, Bhaskar Reddy Yerolla, Ramavath Krishna, Ananya Pandey, Gopinath Ramachandran

**Affiliations:** 1 ESIC Medical College and Hospital, Hyderabad, Telangana, India.; 2 AIIMS Bibinagar, Hyderabad, Telangana, India.

**Keywords:** pancreatitis, pseudocyst, pseudoaneurysm, splenic artery, visceral aneurysm, dsa digital subtraction angiography, pancreatite, pseudocisto, pseudoaneurisma, artéria esplênica, aneurisma visceral, angiografia por subtração digital

## Abstract

Splenic artery pseudoaneurysm is the most common of all the visceral artery pseudoaneurysms. Presentation is often variable and the condition demands immediate diagnosis and management because pseudoaneurysm rupture increases morbidity and mortality. It is associated with pancreatitis and other conditions like abdominal trauma, chronic pancreatitis, pseudocyst of the pancreas, liver transplantation, and, rarely, peptic ulcer disease. We present a case of a giant splenic artery pseudoaneurysm measuring 14x8 cm. Proximal and distal control of the vessels could not be achieved during the procedure because of local adhesions and inflammation and it was necessary to cross clamp the supraceliac aorta to control bleeding.

## Resumo

O pseudoaneurisma de artéria esplênica é o mais comum entre os pseudoaneurismas de artérias viscerais. A apresentação geralmente varia e requer diagnóstico e tratamento imediatos, pois a ruptura do pseudoaneurisma aumenta a morbimortalidade. Esse tipo de pseudoaneurisma está associado à pancreatite e a outras condições, como trauma abdominal, pancreatite crônica, pseudocisto de pâncreas, transplante de fígado e, raramente, úlcera péptica. Apresentamos um caso de pseudoaneurisma gigante de artéria esplênica, com tamanho de 14x8 cm. Durante o procedimento, não foi possível alcançar controle proximal e distal dos vasos devido a aderências locais e inflamação, sendo necessário o clampeamento da aorta supracelíaca para controle do sangramento.

## INTRODUCTION

Splenic artery pseudoaneurysm (SAP) occurs in association with pancreatitis, abdominal trauma, liver transplantation, and, rarely, peptic ulcer disease.^1^ Presentation can vary and pseudoaneurysms have even been detected in asymptomatic patients. Pseudoaneurysms associated with pseudocyst have a greater likelihood of rupture, leading to life-threatening heamorrhage.^2^^,^^3^ Contrast-enhanced computed tomography is the investigation of choice and digital subtraction angiography (DSA) can be used as a diagnostic as well as therapeutic approach to splenic artery aneurysm, depending upon the size of the aneurysm.

## CASE DESCRIPTION

**Figure 1:**
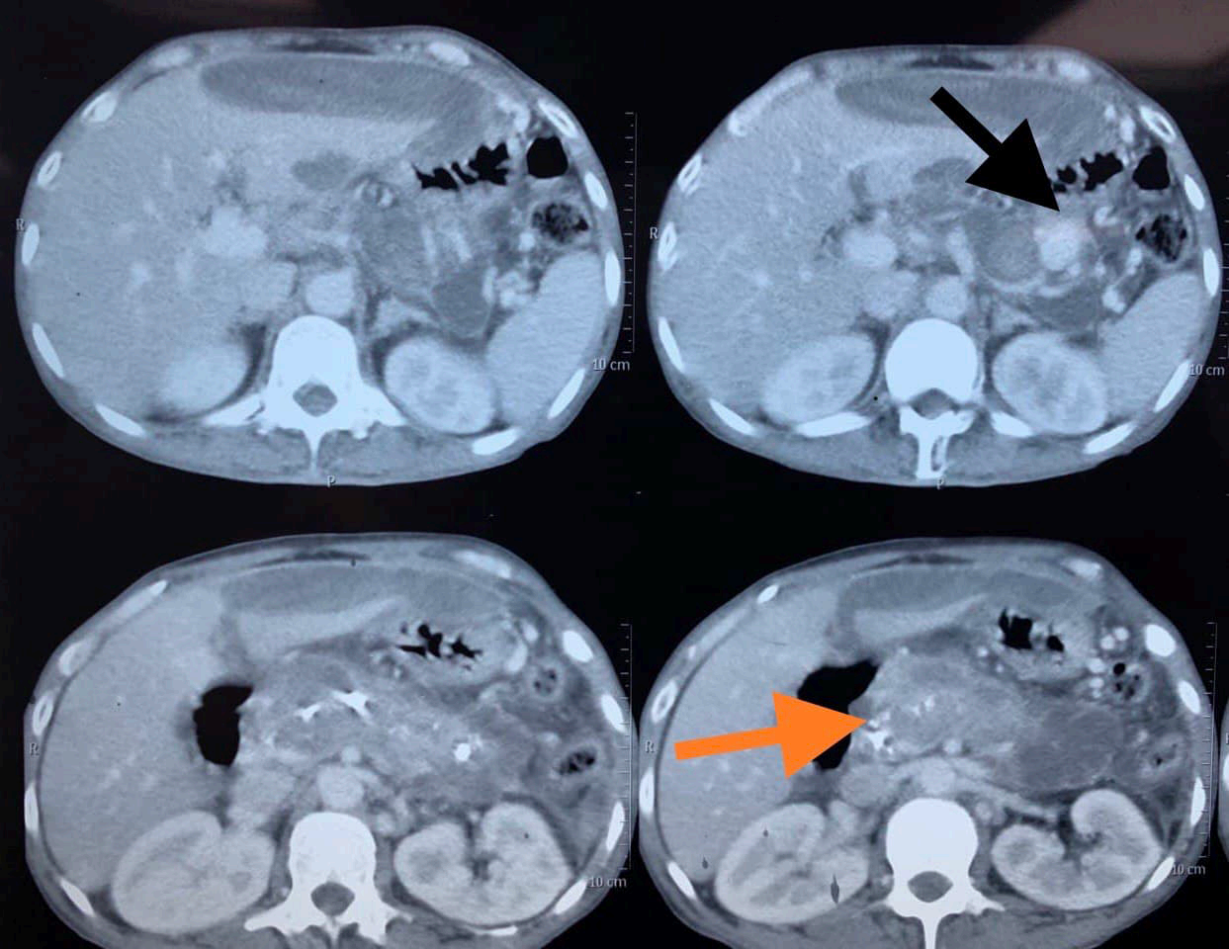
Axial sections from the contrast-enhanced computed tomography (CECT). Black arrow: splenic artery pseudoaneurysm arising from the distal third of the artery. Orange arrow: calcifications within the pancreatic parenchyma.

**Figure 2:**
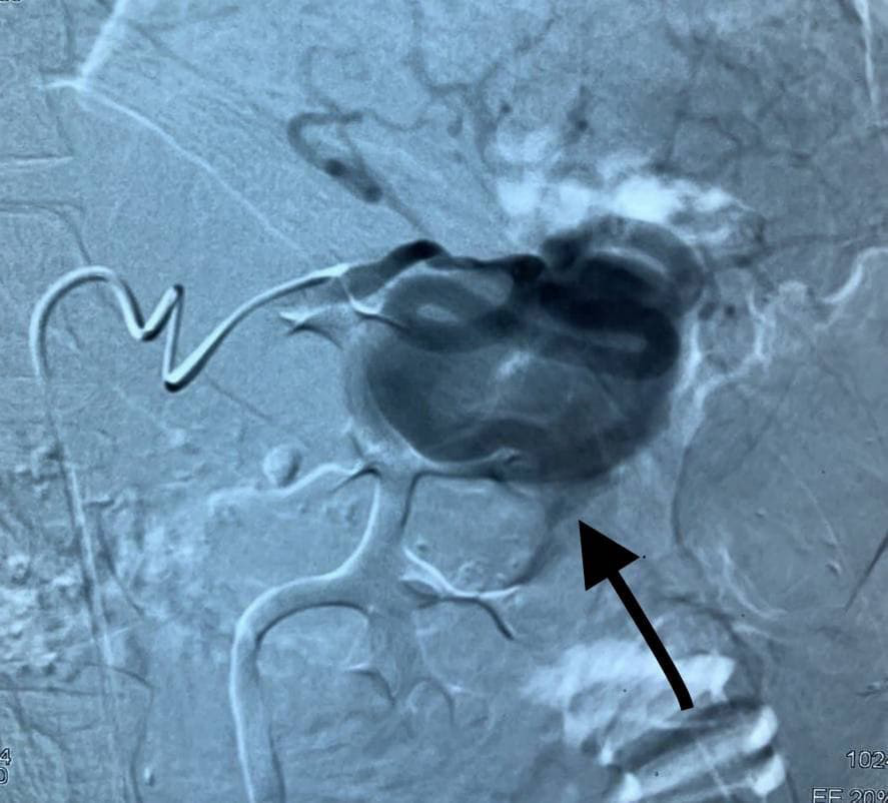
Digital subtraction angiography showing the pseudoaneurysm along with its feeding vessels, which were identified and coil embolized.

**Figure 3:**
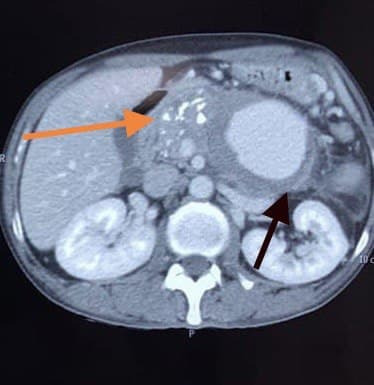
Post coil embolization abdominal CECT showed a giant splenic artery pseudoaneurysm which was still active. Black arrow: giant pseudoaneurysm. Orange arrow: calcifications.

A 50-year-old male with a previous history of chronic pancreatitis came to the hospital with three episodes of melena and epigastric fullness. On clinical examination, he had anemia and his blood pressure was 90/60 mmHg. After resuscitation, he underwent upper gastrointestinal endoscopy which was normal. He also had a history of hematemesis for which he was evaluated with triphasic abdominal contrast-enhanced computed tomography (CECT) (Figure 1). His abdominal CECT showed an 8x6 cm pseudocyst in the lesser sac with a 4x3 cm hyperdense area and multiple calcifications were observed in the pancreas. For the SAP, he underwent digital subtraction angiography embolization and celiac trunk angiography was performed during the procedure by puncturing the femoral artery using the Seldinger method. The splenic artery pseudoaneurysm was identified and a total of three coils were used for embolization (2 coils proximally and 1 coil distally) and no stents were used (Figure 2). Three weeks after embolization, the patient presented with hematemesis and hypovolemic shock. His abdominal CECT showed a giant splenic artery pseudoaneurysm with calcifications in the pancreatic parenchyma (Figure 3). The patient was resuscitated and prepared for exploratory laparotomy, given his clinical condition.

**Figure 4:**
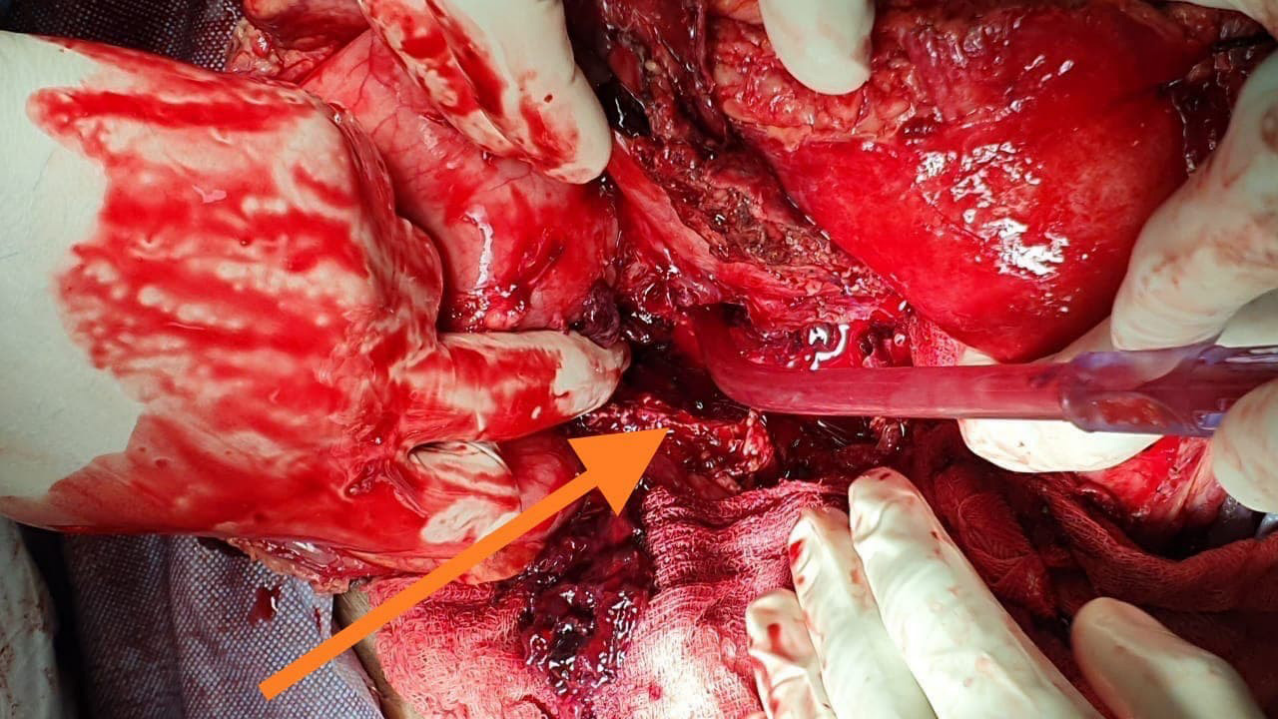
Intraoperative image showing a thrombosed SAP sac, which was opened, and clots were evacuated.

**Figure 5:**
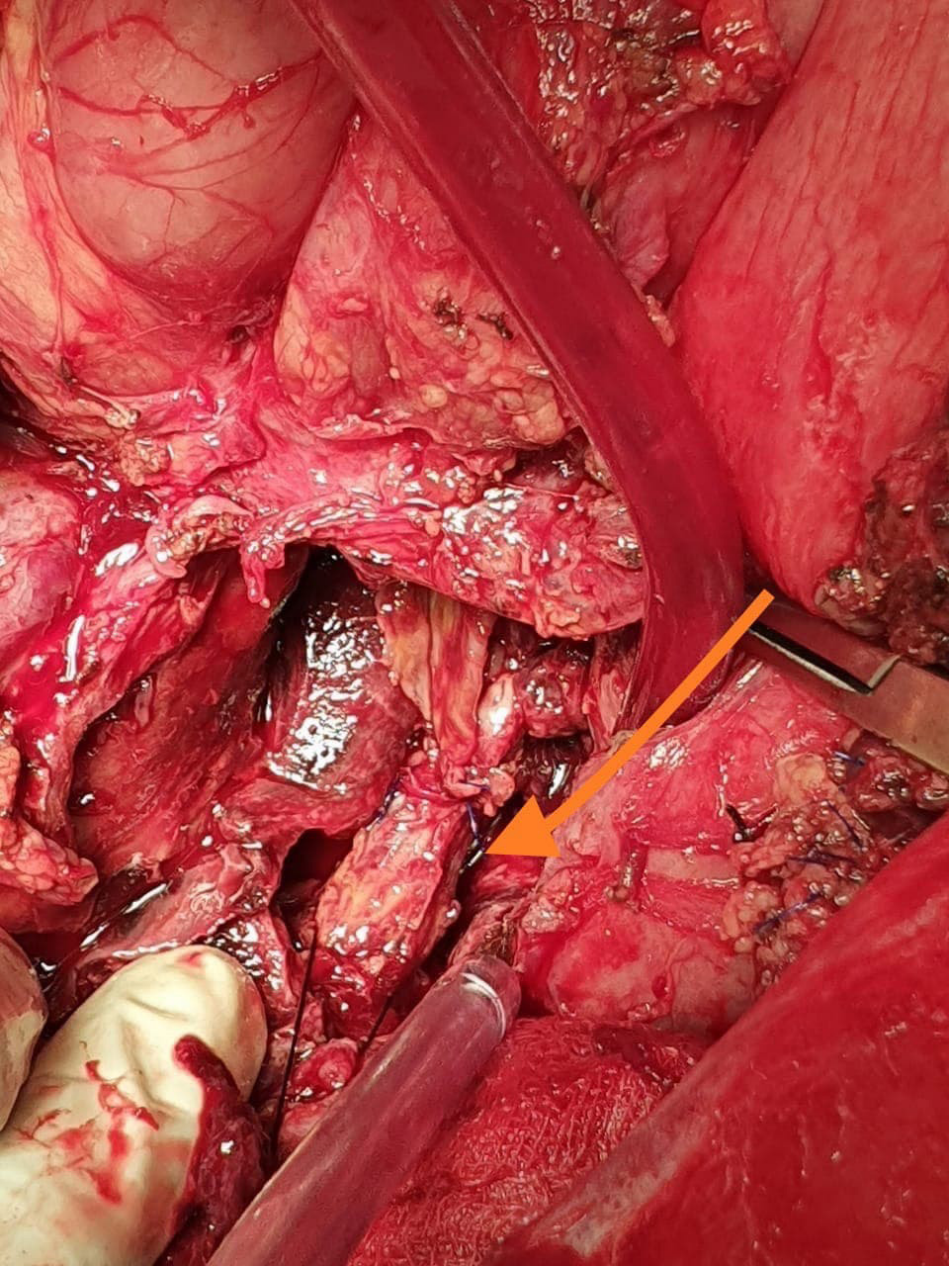
Intraoperative image showing the thrombosed splenic vein, which was identified and ligated.

On exploration, the patient’s abdomen was filled with blood and clots, which were suctioned out. Opening the gastrocolic omentum revealed a thrombosed sac with diffuse bleeding visible from the outer wall of the sac, but no arterial spurt was identified from the sac. After opening the lesser sac, control of the supraceliac aorta was taken, but we were unable to reach the celiac axis and splenic artery proximally or distally because of dense adhesions. After obtaining supraceliac aorta control, we directly opened the sac and an arterial spurt was identified and ligated (Figure 4). Since the splenic vein was thrombosed, it was identified and ligated (Figure 5), followed by splenectomy. Pathology of the pancreas was suggestive of chronic pancreatitis. Postoperatively, the patient had a grade A pancreatic fistula which was managed conservatively and he was discharged on day 7. Anticoagulation medications were not used postoperatively. Six months post-surgery he was asymptomatic.

This manuscript is in accordance with the Helsinki declaration and with local ethical guidelines. Informed consent was obtained from the patient. Written informed consent was obtained from the patient’s husband for publication of this case report and any accompanying images.

## DISCUSSION

Giant pseudoaneurysm of the splenic artery is a rare condition.^3^ A giant pseudoaneurysm is defined as a pseudoaneurysm greater than or equal to 5 cm in size. Giant splenic artery aneurysm has a 28% lifetime risk of rupture. It is a life-threatening condition. General underlying causes of splenic artery pseudoaneurysms are abdominal trauma, chronic pancreatitis, pseudocyst of the pancreas, liver transplantation, and peptic ulcer disease.^4^ The most common presentation of pseudoaneurysms of the splenic artery is upper abdominal pain. Other symptoms are hematemesis, melena, vomiting, and weight loss.

In the index case, the patient came to an outpatient department with a history of prior pancreatitis, presenting with upper abdominal pain and two episodes of melena and gradual epigastric fullness. He was hemodynamically unstable at presentation. Up to 50-58% of patients with SAP can present in a hemodynamically unstable condition. Bleeding can occur in the lesser sac, stomach, pancreatic duct, and retroperitoneum. Ruptured SAP are associated with high rates of mortality and morbidity.^5^ Diagnostic modalities for splenic artery aneurysms include Doppler ultra-sound, abdominal CECT, and magnetic resonance imaging (MRI) angiography.^6^ Computed tomography angiography is useful for observing the typical aneurysm body in the arterial phase. It is also useful for differentiating splenic artery aneurysms from pancreatic tumors, pseudocysts, solid epithelial tumors, and gastric leiomyomas. Magnetic resonance imaging has greater sensitivity and specificity, but it is expensive and will take a longer time in emergency situations. Normally, asymptomatic splenic artery aneurysms are less than 2 cm in size can be followed up. However, all symptomatic pseudoaneurysms of the splenic artery should be managed immediately, either with an endovascular procedure or an open surgical procedure.^7^ Management of splenic artery aneurysms depends on their dimensions and location and on the severity of the clinical findings and the patient’s condition. Digital subtraction angiography embolization is the modality of choice for both diagnosis and treatment.^8^ Nowadays, most cases of splenic artery pseudoaneurysm in patients who are hemodynamically stable are managed by coil embolization. Other modalities are open abdominal surgery or laparoscopic surgery, depending upon patient condition and availability of expertise.

Encouraging results of endovascular treatment and minimal complications indicate endovascular procedures as first-line treatment. However, this method may not suffice in cases with giant pseudoaneurysms.^9^ In cases with recurrent bleeding or after an unsuccessful intervention, immediate aggressive surgery with resection of the spleen and pancreatic tail is indicated.^10^^,^^11^ Hsu et al.^12^ observed that the bleeding rates are high with embolization in chronic pancreatitis patients. Surgery should be considered when DSA embolization fails.

In the index case, the patient was managed with an open surgical procedure. Ideally, one must take control of the splenic artery proximally and distal to the aneurysm. However, in cases with pancreatitis, this may be difficult to achieve because of dense adhesions and sometimes the lesser sac may be frozen due to dense adhesions. In the index case, proximal and distal control of the splenic artery and celiac axis could not be achieved, so we took control of the supraceliac aorta and opened the SAP sac. The main limitation of this case study was non-availability of follow-up images.

## CONCLUSION

Giant pseudoaneurysm of the splenic artery is a rare condition that can occur secondary to pancreatitis. In hemodynamically stable patients, endovascular treatment modalities like coil embolization, stenting, and glue embolization can be tried depending upon the size of the pseudoaneurysm and patient condition, but when an endovascular procedure fails, exploratory laparotomy is the only option to treat the condition. However, in patients with pancreatitis, proximal and distal control may not be possible and in such cases control of the celiac axis or aorta might be helpful.
